# Emerging Therapies for the Management of Pain and Vaso-Occlusive Crises in Patients With Sickle Cell Disease: A Systematic Review of Randomized Controlled Trials

**DOI:** 10.7759/cureus.38014

**Published:** 2023-04-23

**Authors:** Michael Lowe, Zarna Bambhroliya, Hesha Patel, Vishva J Patel, Sunil Akshara Vudugula, Naga Pratyusha Cheruvu, Shafaat Raza, Oluwasemilore I Okunlola

**Affiliations:** 1 Seeking Anesthesiology, Ross University School of Medicine, Fort Lauderdale, USA; 2 Research, California Institute of Behavioral Neurosciences & Psychology, Fairfield, USA; 3 Internal Medicine, Capital Health Medical Center, New Jersey, USA; 4 Medicine, Gujarat Medical Education and Research Society (GMERS) Medical College, Vadodara, IND; 5 Internal Medicine, Jagadguru Sri Shivarathreeshwara Medical College, Mysore, IND; 6 Medicine, Gandhi Medical College, Hyderabad, IND; 7 Medicine, California Institute of Behavioral Neurosciences & Psychology, Fairfield, USA

**Keywords:** pain management, therapy, vaso-occlusive crisis, acute pain, sickle cell crisis

## Abstract

Sickle cell disease (SCD) is an inherited disorder that impairs red blood cells (RBCs) and disrupts the delivery of oxygen to tissues. There is currently no cure. Symptoms can appear as early as six months of age and include anemia, acute episodes of pain, swelling, infections, delayed growth, and vision problems. A growing number of therapies are being investigated for reducing these episodes of pain, also known as vaso-occlusive crises (VOCs). The research literature evidence, however, currently includes far more approaches that have not shown superiority versus placebo than ones that have been proven effective. The purpose of this systematic review is to evaluate the body of randomized controlled trials (RCTs) to determine the quality of support for and against the use of a variety of current and emerging therapies for treading SCD VOCs. Several important new papers have emerged since previous systematic reviews with similar objectives were published. This review was conducted according to the PRISMA (Preferred Reporting Items for Systematic Reviews and Meta-Analyses) guidelines and focused on PubMed exclusively. Only RCTs were sought, and no other filters, except for a five-year historical timeline cut-off, were used. Of the 46 publications that were returned in response to the query, 18 were ultimately accepted as meeting the pre-established inclusion criteria. The Cochrane risk-of-bias tool was utilized as a quality assessment measure, and the GRADE (Grading of Recommendations, Assessment, Development, and Evaluations) framework was used to assess the certainty of the evidence. Among the included publications, five out of 18 featured positive results with superiority and statistical significance versus placebo for either reduction in pain score or number/duration of VOCs. The approaches featured therapies ranging from de novo molecules to currently available drugs approved for other indications to naturally occurring metabolites such as amino acids and vitamins. A single therapy, arginine, was supported for both clinical endpoints: pain score reduction and shortened VOC duration. Currently, two therapies are approved by the United States Food and Drug Administration (FDA) and are commercially available (crizanlizumab, ADAKVEO and L-glutamine, Endari). All other therapies are investigational only in nature. Several studies included measurement of biomarker endpoints as well as clinical outcomes. Generally, beneficial outcomes related to improving biomarker levels did not also translate into statistically significant reduction of pain scores or number/duration of VOCs. While measuring biomarkers may contribute to the understanding of pathophysiology, it does not appear to directly offer predictive value toward treatment success clinically. It can be concluded that there exists a specific opportunity to design, fund, and execute investigations that both compare emerging and existing therapies versus one another and compare combinational therapies versus placebo.

## Introduction and background

Pain and vaso-occlusive crises in patients with sickle cell disease

**Figure 1:**
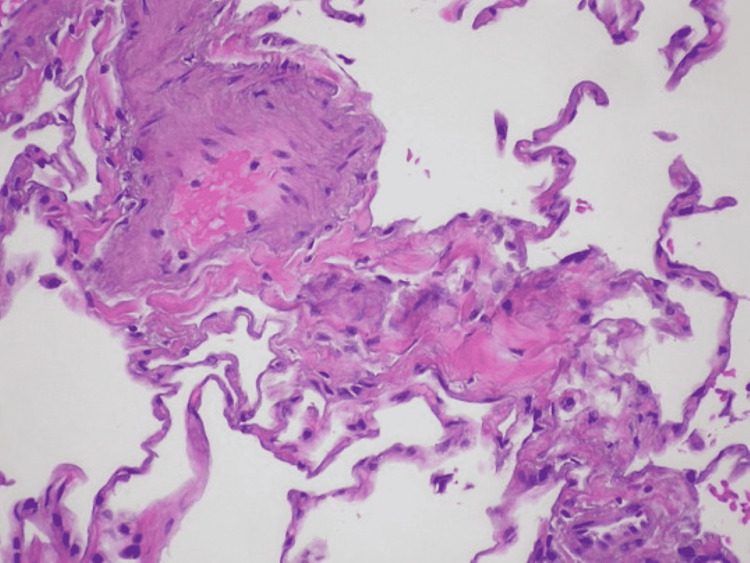
Microanatomy of vaso-occlusive disease Arteriolar obstruction associated with pulmonary veno-occlusive disease (PVOD) - Case 269 (8655564066).jpg [3]. Licensed under the Creative Commons Attribution-Share Alike 2.0 Generic license.

Sickle cell disease (SCD) is an inherited red blood cell (RBC) disorder that currently has no cure. The epidemiology of SCD is significant, with around 100,000 new cases/infant births per year in the United States and another 200,000 worldwide [1]. One of the most feared and concerning complications of SCD is the vaso-occlusive crisis (VOC) episode. VOCs occur when deformed RBCs, with the characteristic "sickle" shape, occlude blood flow. In severe instances, this occlusion can lead to oxygen deprivation. As a compensatory mechanism, the body initiates an inflammatory response [2]. The downstream result of this response is significant and acute pain, which most commonly occurs in the extremities, although it is also frequently reported in the chest and back regions. This occlusion is also known to cause pulmonary vasculature pathology. Exemplary histology of such vessel occlusion can be seen in Figure 1.

**Figure 2:**
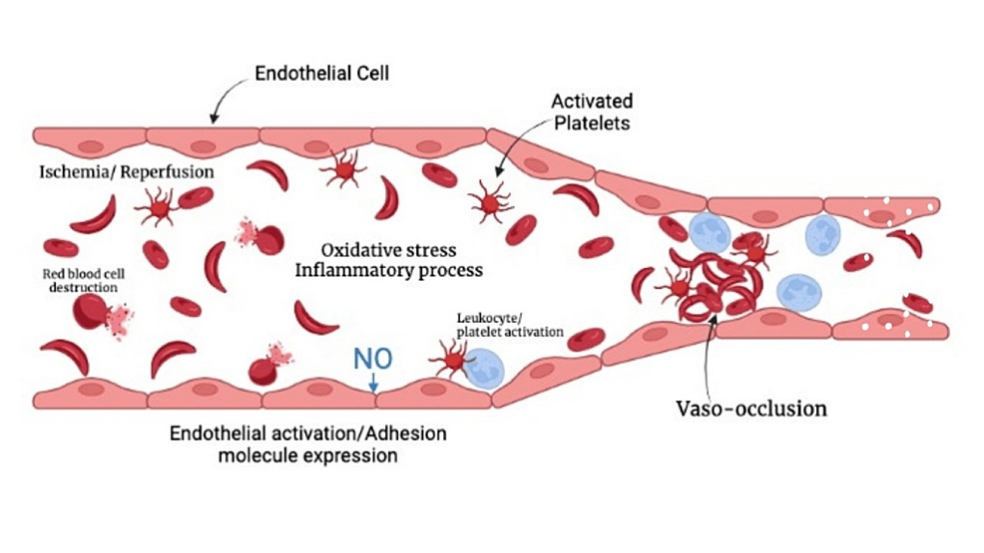
Pathology of arterial vaso-occlusion Illustration is original work of authors (M. Lowe, Z. Bambhroliya) NO, nitric oxide

A schematic demonstrating the mechanical and pathological nature of the occlusive event is seen in Figure 2.

Current approaches

There have been a variety of clinical treatment approaches to the management of these pain crisis episodes in patients, but with no clearly superior therapy emerging. Therapeutic agents deployed include naturally occurring organic compounds such as amino acids arginine [4], L-glutamine (GLN) [5], amino acid derivative N-acetylcysteine (NAC) [6], vitamin D [7-8], and even cannabis [9]. In addition, a number of biopharmaceuticals have been used, including pregabalin [10], montelukast [11], voxelotor [12], crizanlizumab [13], morphine [14-16], ketamine [14-15], and ticagrelor [17]. Finally, not surprisingly, pain relievers such as diclofenac [18] and acetaminophen [18-19] have been routinely prescribed.

Cooper et al. previously published a review on pharmacological interventions for sickle cell VOC pain [20]. This well-written review is now nearly seven years old. In that time, a number of trials and resulting publications became available, and many of them were related to new therapeutic options that did not exist in 2016. Additionally, Cooper et al. limited their review to patients of age 18 or older only. The current body of literature also includes meta-analysis publications that explored a single therapy, including all of the available evidence of any format for that specific given therapy. Examples include review studies that more deeply investigated crizanlizumab [21] and ketamine [22]. The crizanlizumab review ultimately analyzed only two of the 52 records that remained following duplicate removal. Furthermore, only 11 of the 52 were published. The ketamine review also included a number of less-stringent publication formats: of the 14 studies, only one was a clinical trial. Other records included five case reports, four case series, and three single-arm observational studies.

In 2014, an expert panel was brought together to publish consensus guidelines to support the management of SCD [23]. The scope of the mission and vision of this panel is explained clearly in its report. SCD is a rare clinical condition. For this reason, the number of healthcare providers who are capable of and prepared to deliver expert care or consultation is very small.

Our objective was to identify and evaluate randomized controlled trials (RCTs), published in peer-reviewed journals, that investigated existing and emerging therapies for treating SCD patients at risk of VOC. Findings are of interest to healthcare professionals, patients and their families and caregivers, the insurance industry, pharmaceutical manufacturers, and compliance regulating authorities.

## Review

Methods

We searched for RCTs in which patients presenting with SCD experiencing VOC were treated therapeutically. This review was conducted according to the PRISMA (Preferred Reporting Items for Systematic Reviews and Meta-Analyses) guidelines. A historical timeline filter of five years was used in an effort to focus on the most recent and emerging therapies, as well as to build on previous reviews, as opposed to duplicate them. There were no filters used for study location, single center versus multicenter, setting, or patient population characteristics, including gender, age, race/ethnicity, or nationality. All articles screened were available in English; and only articles readily available in full-text format were selected. Upon review, the patient profile was verified as SCD. There were no deviations from the pre-established strategy. The date of the last search was December 31, 2022, and results were verified via independent co-author validation.

PubMed

The following keywords were used: “Sickle Cell Disease,” “Pain,” “Treatment.” As filters, publication type selected was “Randomized Controlled Trial” and publication date selected was "5 years" selected; all other fields in all other categories deselected.

The Cochrane risk-of-bias assessment tool was used [24]. Articles were included regardless of the final assessment because there is a paucity of well-designed research on this topic.

Results

**Figure 3:**
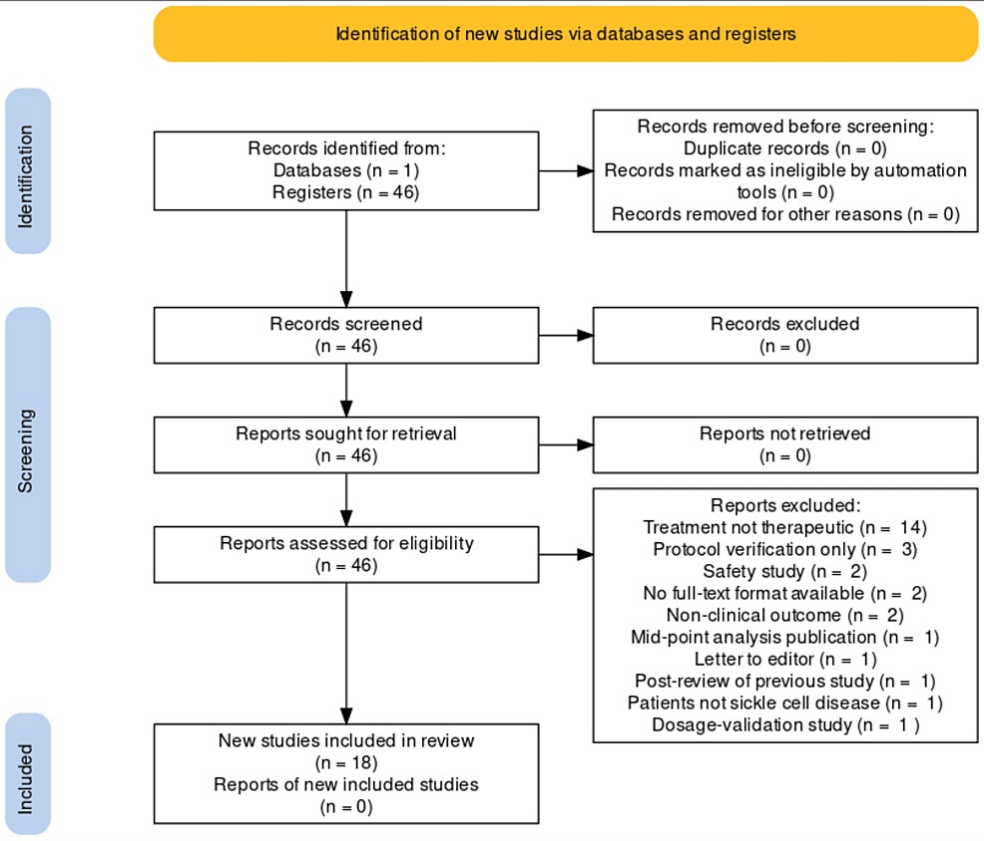
PRISMA flow diagram Image credit: Original work of authors (M. Lowe) PRISMA, Preferred Reporting Items for Systematic Reviews and Meta-Analyses

A total of 46 citations were returned from PubMed and assessed, with 18 ultimately reviewed (Figure 3). 

**Figure 4:**
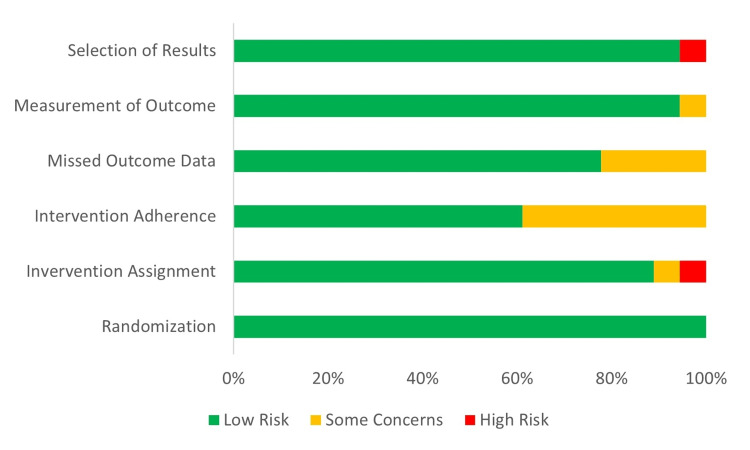
Cochrane risk-of-bias summary Image credit:  Original work of authors (M. Lowe)

In assessing studies for eligibility, 28 of the 46 records were excluded for a variety of reasons. Many of the RCTs were well-designed and of clinical importance to SCD but did not investigate therapeutic treatments. Examples included the use of mobile device apps, multimedia education tools, pain scale development, and even medical devices for airway management. There were two that did not investigate clinical outcomes (one measured sub-cellular mitochondrial reaction pathway products and another measured macrophage markers). Finally, one study featured patients diagnosed with thalassemia, not SCD. The risk of bias summary is included in Figure 4.

The details of the included studies are summarized in Table 1, including date, location, size, therapy investigated, design, endpoint, and conclusion.

**Table 1:** Summary of randomized controlled trials assessed VOC, vaso-occlusive crisis; # VOCs, number of VOC episodes; HRQL, health-related quality of life

Trial	Year	Country	n	Treatment	Control	Endpoint category	Conclusion
Niihara et al. [5]	2018	United States	230	L-glutamine	Placebo	# VOCs	Pain crises over 48 weeks was lower with L-glutamine vs. placebo, regardless of hydroxurea use
Abrams et al. [9]	2020	United States	23	Cannabis	Placebo	Pain Scores	No statistically significant difference in pain between cannabis and placebo
Kutlar et al. [13]	2018	United States	198	Crizanlizumab	Placebo	# VOCs	35.8% of crizanlizumab patients did not experience a VOC vs. 16.9% of placebo patients
Casella et al. [25]	2021	66 sites in 12 countries	388	Poloxamer 188	Placebo	Time to VOC	Poloxamer 188 did not significantly shorten time to opioid use for VOC
Schlaeger et al. [10]	2017	United States	22	Pregabalin	Placebo	Pain scores	Mean pain scores were no different between pregabalin and placebo
Field et al. [11]	2020	United States	41	Montelukast	Placebo	Pain scores	No difference between montelukast and placebo groups
Lubega et al. [14]	2018	Uganda	240	Morphine, ketamine	Comparative	Pain scores	Time to achieve maximum pain score reduction was 19.8 minutes for ketamine vs. 34.1 minutes for morphine
Glassberg et al. [26]	2017	United States	54	Inhaled Steroids	Placebo	Pain scores	Mometasone statistically significantly reduced daily pain scores by 1.42 points
Kanter et al. [17]	2018	United Kingdom	87	Ticagrelor	Placebo	Pain scores	No effect on pain scores was detected
Dougherty et al. [7]	2020	United States	44	Vitamin D	Comparative doses	Pain scores	Improvement for both groups in HRQL scores, of which pain is a component
Panda et al. [18]	2019	India	104	Acetaminophen, diclofenac	Comparative	Pain scores	Intravenous acetaminophen is a better alternative to intravenous diclofenac
Daak et al. [27]	2018	United States	62	SC411	Comparative doses	# VOCs	The rate of sickle cell crisis was 53% lower for the pooled active groups vs. placebo
Onalo et al. [4]	2021	Nigeria	68	Arginine	Placebo	Time to VOC	Arginine resulted in more rapid pain control, reduced analgesic load requirement, and improved time-to-crisis
Gregoire-Pelchat et al. [8]	2020	Canada	38	Vitamin D	Placebo	# VOCs	No difference between Vitamin D and placebo group
Sins et al. [6]	2019	11 sites Belgium, United Kingdom, Netherlands	67	N-acetylcysteine	Placebo	# VOCs	Treatment with N-acetylcysteine was not of clinical benefit vs. placebo
Alshahrani et al. [15]	2021	Saudi Arabia	278	Morphine, ketamine	Comparative	Pain scores	Ketamine resulted in a reduction in pain scores over a two-hour period and decreased morphine dose requirement
Quarrie et al. [16]	2020	United States	80	Warm saline with morphine	Vs. cold saline with morphine	Pain scores	Warm saline resulted in no difference in pain scores or morphine requirement
Dhebaria et al. [19]	2022	United States	81	Acetaminophen	With Morphine vs. morphine alone	Pain scores	Acetaminophen resulted in no difference in pain scores or morphine sparing

Careful consideration was given to the best way to organize these studies in order to assess the entire body of research and draw conclusions. The decision was made to group the publications based on the endpoint being measured. The 18 publications that were assessed can be categorized as follows.

There were five RCTs with the endpoint designed as, "number of vaso-occlusive crisis episodes." These five RCTs investigated five different therapies. There were over twice as many RCTs (11 in total) with the endpoint designed as, "pain score reduction." These 11 RCTs investigated nine different therapies (with ketamine vs. morphine, and acetaminophen studied in two different publications each). Finally, there were two publications with, "time to VOC," as the endpoint. One of these two (arginine) was also measured for pain score reduction, and will therefore show up multiple times in this review. We will begin with the results of this last category.

The contest for a reduced "crisis time" benefit is not difficult to interpret and does not require analysis beyond superficial assessment of conclusions. Simply put, Casella et al. reported no crisis episode time/duration benefit with poloxamer 188 [25], while Onalo et al. did report a benefit using arginine [4].

Poloxamer 188 is classified chemically as a nonionic block polymer surfactant. Previously, it has been reported to improve blood flow in microvessels via reducing cell-cell interaction as well as blood viscosity [28]. It also makes a logical candidate for therapy given its track record. It was previously evaluated in three separate SCD trials demonstrating safety and possible efficacy [29-32]. These publications investigated not only VOCs but also acute chest syndrome, which often arises as a result of intrapulmonary vascular occlusion. In the RCT assessed for this current systematic review, however, poloxamer 188 failed to deliver promising results. Casella et al. administered an IV loading dose (100 mg/kg) followed by continuous infusion (30 mg/kg/hour for up to 48 hours) and compared the results to a matched placebo arm (each with n=194). The treatment arm results were no different from placebo in terms of the VOC timeframe (actually measured as time to last dose of parenteral opioids during a vaso-occlusive episode) [25].

Using this same endpoint, however, arginine was shown by Onalo et al. to result in a benefit. Children receiving arginine (n=35) experienced a shorter time-to-crisis resolution (p=0.02) versus the matched placebo arm (n=33). At the 50-hour post-crisis onset time point, only 20 arginine-treated patients remained unresolved versus 27 in the placebo group. The practical significance of this benefit cannot be overstated. The arginine-treated patients were also documented as having a shorter hospital stay (105 hours in total vs. 141 hours for placebo, p=0.002) and experienced no serious adverse events.

In summary, the benefit of reduced crisis episode duration was measured using poloxamer 188 and arginine. Interestingly, both RCTs were published in 2021. Only arginine showed a benefit, and these results are summarized in Table 2.

**Table 2:** Randomized controlled trials with time/duration-of-crisis benefit as endpoint JAMA, Journal of the American Medical Association

Trial/publication	Year	Journal	n	Treatment	Methodology to measure duration	Summary conclusion
Casella et al. [25]	2021	JAMA	388	Poloxamer 188	Time to opioid use	No statistically significant difference between treatment and control
Onalo et al. [4]	2021	Am J Hematol	68	Arginine	Time to resolution	Treatment was effective

Second, looking now at the RCTs investigating number of VOCs (as opposed to reduced duration of episodes), there are more data to review. The five studies that shared this endpoint are summarized in Table 3.

**Table 3:** Randomized controlled trials with number of vaso-occlusive crisis episodes experienced as endpoint

Trial/publication	Year	Journal	n	Treatment	Summary conclusion
Niihara et al. [5]	2018	N Engl J Med	230	L-glutamine	Treatment was effective
Kutlar et al. [13]	2018	Am J Hematol	198	Crizanlizumab	Treatment was effective
Daak et al. [27]	2018	Blood Advances (The American Society of Hematology)	62	SC411	No statistically significant difference between treatment and control
Gregoire-Pelchat et al. [8]	2020	British Journal of Haemotology	38	Vitamin D	No statistically significant difference between treatment and control
Sins et al. [6]	2019	British Journal of Haemotology	67	N-acetylcysteine	No statistically significant difference between treatment and control

Of the five RCTs, it can be seen that two of the treatment therapies (GLN, crizanlizumab) were found to be effective. Another two of the treatment therapies (SC411, NAC) were found to be effective but did not reach statistical significance. Finally, one of the treatment therapies (vitamin D) was found to be no different or better than placebo.

Although Gregoire-Pelchat et al. did not demonstrate a benefit with vitamin D in the study assessed in this review [8], this same research group had previously identified an interesting trend. In tracking SCD children through a large tertiary care center hospital in Montreal, Canada, a high vitamin D deficiency rate (<50 nmol/L) was observed [33]. They further noted that both dietary intake and supplementation were low in the investigation setting.

Daak et al. demonstrated impressive improvement across several biomarkers using SC411 but failed to achieve statistical significance with the clinical outcomes [27]. SC411 is a docosahexaenoic acid (DHA) ethyl ester formulation. It is a logical therapeutic candidate based on the fact that SCD patients typically have low RBC levels of DHA as well as eicosapentaenoic acid (EPA) [34]. Indeed, patients who took a 383-mg SC411 soft gel mini-capsule once daily demonstrated improved levels of RBC DHA, RBC EPA, D-dimer, E-selectin, and even hemoglobin when compared to a matched cohort of patients who took a soybean oil placebo pill [27]. In terms of pragmatic benefit and clinical importance, however, hospitalization rate, hospitalization days, medical facility visits, and school day absences did not achieve statistically significant differences between treatment and placebo groups. Of note, a reduction in the rate of crisis episodes was observed in the pooled treatment groups versus placebo, but it also did not reach statistical significance (p=0.07) [34].

The interest of Sins et al. in NAC was based on the antioxidant's previous merits with SCD patients. It had been previously shown in the literature to improve biomarkers on oxidative stress and even hemolysis [35-36]. From an access-to-care perspective, NAC is generally recognized as safe and is also inexpensive. In this particular study, 40 placebo patients reported an event rate per year of 28.5 (extrapolated from days with VOC) compared to only 27.4 from the 27 NAC patients [6]. Aside from this negligible benefit difference, the finding also lacked statistical significance (p=0.94). Compliance was measured in terms of returned medication bottles and tablet counts, and it was noted that more NAC patients reported gastrointestinal issues, which may explain the higher drop-out rate.

Conversely, the two studies that did feature favorable results with statistical significance in terms of number of VOCs experienced included GLN and crizanlizumab. Pharmaceutical-grade GLN, a charge-neutral, polar amino acid, was administered by Niihara et al. at a dose level of 0.3 mg/kg of body weight to 152 patients [5]. The results were compared to 78 placebo patients who received 5 g of white, unflavored powder. Over 48 weeks, the GLN group reported only three discrete pain crisis episodes versus four in the placebo group (p=0.005). It should be noted that the difference in scale here with Niihara et al. versus Sins et al. is because the former measured discrete crisis episodes, while the latter measured a number of days (a single VOC episode can last for more than one day). It should be noted that two-thirds of the patients in each arm of Niihara et al.’s RCT received concomitant hydroxyurea [5].

Crizanlizumab has been described in the literature as a humanized monoclonal antibody that exhibits anti-P-selectin preference [37]. In this trial, crizanlizumab (5 mg/kg) was administered 14 times over the course of a full year (including two loading doses in month one, with subsequent monthly infusion) to 66 patients [13]. The control arm comprised of 67 placebo patients. Subgroup analysis suggested that the annual rate of VOCs occurred with a lower frequency in patients using crizanlizumab versus placebo regardless of hydroxyurea use, SCD genotype, or historical number of VOCs. It is very important to note that there was a third arm, with a lower-dosage crizanlizumab therapy of 2.5 mg/kg. The post-hoc analysis only evaluated the effect of the 5 mg/kg crizanlizumab dosage level. The evaluation of 2.5 mg/kg crizanlizumab failed to meet the primary endpoint.

Third, in turning focus to the studies which that benefit as pain score reduction, the largest number of studies can be observed. They are summarized in Table 4.

**Table 4:** Randomized controlled trials with reduction in pain score as endpoint JAMA, Journal of the American Medical Association

Trial/publication	Year	Journal	n	Treatment	Summary conclusion
Abrams et al. [9]	2020	JAMA	23	Cannabis	No statistically significant difference between treatment and control
Schlaeger et al. [10]	2017	Pain Management Nursing	22	Pregabalin	No statistically significant difference between treatment and control
Field et al. [11]	2020	Blood Advances (American Society of Hematology)	41	Montelukast	No statistically significant difference between treatment and control
Lubega et al. [14]	2018	Scand J Pain	240	Morphine, ketamine	No statistically significant difference between treatment and control
Glassberg et al. [26]	2017	Am J Hematol	54	Inhaled steroids	Treatment was Effective
Kanter et al. [17]	2018	British Journal of Haemotology	87	Ticagrelor	No statistically significant difference between treatment and control
Dougherty et al. [7]	2020	J Pediatr Health Care	21	Vitamin D	Treatment was effective
Panda et al. [18]	2019	Indian Pediatrics	104	Acetaminophen, diclofenac	Acetaminophen is effective (relative to diclofenac)
Alshahrani et al. [15]	2021	Acad Emerg Med	278	Morphine, ketamine	No statistically significant difference between ketamine and morphine
Quarrie et al. [16]	2020	Pediatric Emergency Care	80	Warm saline with morphine	No statistically significant difference between treatment and control
Dhebaria et al. [19]	2022	Academic Emergency Medicine	81	Acetaminophen	No statistically significant difference between acetaminophen and morphine
Onalo et al. [4]	2021	Am J Hematol	68	Arginine	Treatment was effective

Of the 12 RCTs that featured reduction in pain score as an endpoint, only three demonstrated a statistically significant benefit: mometasone [26], vitamin D [7], and arginine [4]. A fourth concluded relative superiority of acetaminophen to diclofenac [18]. As a point of emphasis, vitamin D was found to be ineffective in Gregoire-Pelchat et al.’s study [8], but that RCT measured number of VOCs as the benefit, as opposed to reduction in pain scores.

Mometasone, like all corticosteroids, exhibits anti-inflammatory, antipruritic, and vasoconstrictive properties. As a class, corticosteroids reduce allergic reactions in mastocytes and eosinophils. A convincing transitive link has been established in the literature between respiratory symptoms and SCD pain [38]. Glassberg et al. conducted the RCT assessed in this review based on the premise that therapies traditionally used to treat airway obstruction and inflammation could also benefit non-asthmatic SCD patients. Asthma as a risk factor for SCD morbidity has been well-established [39]. In fact, a recent observational study concluded high rates of airway pathology (hyperreactivity, obstruction, and wheezing) in non-asthmatic SCD patients [40]. The results obtained by Glassberg et al. were impressive and straightforward: over a 16-week duration, mometasone patients reported a mean pain diary score of 1.42 points less than (favorable to) the matched placebo arm on a 6-point scale (95% CI: 0.61-2.21, p=0.001) [26].

Dougherty et al. reported significant reductions in pain with administration of vitamin D [7], a notable departure from the other vitamin D study included in this assessment by Gregoire-Pelchat et al. [8]. Dougherty et al. compared two vitamin D3 (cholecalciferol) oral dosage levels (4,000 vs. 7,000 IU) across two matched cohorts: sickle cell disease (HbSS) and healthy subjects. Evaluations were made at six- and 12-week intervals. The PROMIS (Patient-Reported Outcomes Measurement Information System) assessment tool was used. It has been previously validated in the literature [41] and includes "pain impact" scores, among others. From baseline, to six weeks, to 12 weeks, the pooled PROMIS pain impact scores for the HbSS group decreased from 54.4 to 52.7 to 48.4, respectively (p<0.05, six-week levels).

Arginine, the third of the three statistically significant efficacious treatments evaluated with reduction in pain scores as a benefit endpoint, was also effective with a time-to-resolution-of-VOC benefit. Onalo et al. took care in their RCT to match the arginine treatment and placebo arms (n=35, baseline pain score 8.7 ± 1.1 vs. n=33, 8.4 ± 1.3, respectively) [4]. The pain scores for the arginine group improved over time, but unfortunately all of the results are presented visually and not described quantitatively with semantics. It was stated that the average rate of decline for worst pain score was more rapid with arginine therapy (1.50 points/day vs. only 1.09 points/day for the placebo group, p=0.09) [4].

In summary, the result highlights are as follows. A total of 18 RCTs were qualified and analyzed and subsequently divided into three categories based on endpoint: duration to benefit (two trials), number of VOCs (five trials), and reduction in pain scores (12 trials, one of the RCTs was included in two endpoint categories). For duration to benefit, arginine was the only effective therapy [4]. For number of VOCs, GLN and crizanlizumab were effective treatments [5,13]. For reduction in pain scores, mometasone, vitamin D, and arginine were superior to placebo [4,7,26]. In total, across all endpoint categories, five discrete therapies were demonstrated as being efficacious. 

Discussion

Several themes emerged through our research findings. First, SCD presents clinically in a complex way. The measurement of pain and discomfort experienced by these patients can take on a variety of forms and measures. Table 5 features a list of endpoints that have been observed within the research literature.

**Table 5:** Endpoints that have been measured in research literature on patients with sickle cell disease VAS, visual analog scale; ASCQ-Me, Adult Sickle Cell Quality of Life Measurement Information System; NPRS, numeric pain rating scale; NRS, numeric rating scale; RASS, Richmond Agitation-Sedation Scale; VOC, vaso-occlusive crisis; MQS, Medication Quantification Scale; HRQL, Health-Related Quality of Life; PROMIS, Patient-Reported Outcomes Measurement Information System; BOTMP, Brulninks-Oseretsky Test of Motor Proficiency; PEDSQL, Pediatric Quality of Life Inventory; SF, Short Form; RBC, red blood cell; EPA, eicosapentaenoic acid; DHA, docosahexaenoic acid; WBC, white blood cell; sVCAM, soluble vascular cell adhesion molecule; MCV, mean corpuscular volume; IL, interleukin; TNF-a, tumor necrosis factor-alpha; IFN-g, interferon gamma; eNO, eNitric oxide

Efficacy
Pain score reduction
VAS
ASCQ-Me
Brief pain inventory
PAINReportIt
Leeds assessment of neuropathic signs and symptoms
Neuropathic pain symptom inventory
NPRS (also abbreviated as NRS)
Musculoskeletal pain
RASS
Pain diaries
Rate of painful crises
Intensity of painful crises
Time-to-first VOC
Frequency of analgesic use at home
School attendance
Cumulative dose of opioids/morphine equivalents required
Duration to last administration of opioids
Analgesic usage via the analgesic MQS
Duration of hospital stay
Hospital/medical facility admission rate
Hospital readmission rate following discharge
Number of exchange/simple blood transfusions
Incidence of treatment failures
Physiological
Pulmonary function testing
Satisfaction
Survey tools
HRQL scores via PROMIS
Physical functioning scores
Motor proficiency scores via BOTMP
PEDSQL
SF-36 Health Survey
Biomarkers
RBC membrane concentration of EPA and DHA
Serum 25(OH)D
Plasma lactate dehydrogenase
D-dimer
Reticulocyte count
WBC count
Platelets
Indirect bilirubin
High-sensitivity C-reactive proteins
sVCAM
Soluble E-selectin
Soluble P-selectin
Hemoglobin
MCV
Microvascular blood flow
Interleukins (IL-1b, IL-6, IL-4, IL-2, IL-13)
Cytokines (TNF-a and IFN-g)
Change in eNO
Calcium
Urinary calcium/creatinine ratio
Safety
Hemodynamic parameters
Heart rate
Mean arterial pressure
Oxygen saturation
Adverse events
Adverse effects of morphine
Pneumonia
Acute chest syndrome
Death

The implication of having so many different endpoint measurements is that it becomes difficult to directly compare results across studies or apply meta-analysis methods. For example, even though vitamin D, inhaled mometasone, and arginine were found to be effective with regard to a common endpoint of improving pain scores [4,7,26], they still cannot really be directly compared because of the differences across the pain score measurement details. Glassberg et al. deployed the Adult Sickle Cell Quality of Life Measurement Information System® (ASCQ-Me) to investigate mometasone [26]. It is a tool that has been validated and publication-reviewed by HealthMeasures, which is the National Institute of Health's (NIH) measurement resource [42]. It provides measurable attributes for adults with SCD and reports on a 100-point scale. Onalo et al. chose to use a less standardized approach for arginine and simply asked treatment arm and placebo patients to rate their daily pain on a scale of 1 (least pain) to 10 (most pain) [4]. Finally, in their investigation of vitamin D, Dougherty et al. used the PROMIS pediatric short form for pain [7]. PROMIS stands for Patient-Reported Outcomes Measurement Information System, and it has been demonstrated as a valid and reliable measurement tool for SCD patients [43]. Incidentally, this RCT also used the PROMIS short forms for depressive symptoms, fatigue, mobility, peer relationships, and upper-extremity function.

It is not difficult to conceptualize that superior performance using one of these methodological scales does not necessarily translate into a similar outcome with other, even similar, scales. For example, it cannot be known whether or not mometasone would be superior to arginine and vitamin D, if all three therapies were compared using the ASCQ-Me tool, nor if vitamin D would be superior if mometasone and arginine were measured using the PROMIS short form, so on and so forth. There is certainly an opportunity for larger trials that are comparative in nature across two or even three different therapeutic arms.

The second discussion theme is interesting: overall, irrespective of RCT design or endpoint, there are a significantly larger number of therapy candidates that are not efficacious than therapies that are efficacious. A summary listing of the therapies from RCTs assessed in this review, categorized according to effectiveness, can be seen in Table 6 (note that vitamin D appears on both lists, as there were separate RCTs supporting and refuting its effectiveness).

**Table 6:** Proposed therapies for the treatment of sickle cell disease vaso-occlusive crisis categorized by efficacy demonstrated versus no efficacy demonstrated

Therapies shown to be statistically significantly superior to placebo	Therapies shown to be not different vs. placebo with statistical significance
Arginine	Cannabis
L-glutamine	Poloxamer 188
Crizanlizumab	Pregabalin
Mometasone	Montelukast
Vitamin D	Morphine, ketamine
	Ticagrelor
	Vitamin D
	Acetaminophen, diclofenac
	SC411
	N-acetylcysteine
	Warm saline with morphine
	Acetaminophen

The point of this analysis and categorization of the existing and proposed therapies is that a widely accepted, effective treatment for managing VOC pain in SCD patients remains somewhat elusive. There exists an opportunity to cocktail therapies in order to investigate whether or not combinations of these agents, as well as others, may act synergistically.

A discussion around each of the five therapies that did demonstrate superior efficacy versus placebo is warranted. It should be noted that the only two currently approved for use in treating SCD VOCs by the FDA are crizanlizumab (ADAKVEO, Novartis Pharmaceutical Corporation, East Hanover, New Jersey) and GLN (Endari, Emmaus Medical, Inc., Torrance, CA).

Crizanlizumab was approved by FDA on November 15, 2019, making it a somewhat recently available drug [44]. Novartis Pharmaceuticals Corporation manufactures and markets crizanlizumab under the trade name ADAKVEO®. The prescription indications for use (IFU) label reads as follows: "ADAKVEO® is indicated to reduce the frequency of vasoocclusive crises (VOCs) in adults and pediatric patients aged 16 years and older with sickle cell disease [45]." Initial dosing is intravenous (IV) for 30 minutes, followed by two bridging doses two weeks apart, and finally an infusion every four weeks on an ongoing basis.

The primary drawbacks with crizanlizumab currently are two-fold. First, there is only a single RCT that supports its effectiveness. While Kutlar et al. published a very convincing investigation that included 60 sites across three countries [13], the fact remains that there have been no additional studies to date. Second, the IFU is limited to patients aged 16 years and older. Younger pediatric patients, therefore, must be treated off-label.

Arginine therapy for SCD has yet to be tested in phase 3 clinical trials. The paper assessed in this review, Onalo et al., provides the most convincing evidence to date in the form of a phase 2 clinical trial [4]. The premise for the use of arginine is simple. Its metabolism produces nitric oxide (NO), a known potent vasodilator. Increased bioavailability of NO contributes to the prevention and maintenance of coagulopathy through its vasodilatory properties, as well as its platelet aggregation inhibition profile. The benefits resulting from this metabolic pathway are many, such as reduced pulmonary hypertension [46], reduced mortality [47], and reduced pediatric hospitalization [48]. As previously mentioned, it was the only therapy reviewed here that delivered statistically significant favorable results on two endpoints: reduction of both pain scores and time duration to VOC.

The shortcomings with arginine currently include the limitations reported by the authors of this Nigerian-based publication. Namely, only two sites were used for the investigation. Perhaps more importantly, the reproducibility of results in this limited population may be difficult to generalize across broader groups due to the unique medical history often seen with patients in sub-Saharan Africa. For example, both nutritional status and the presence of infectious disease may be difficult variables to control. Both malaria and human immunodeficiency virus (HIV) are known to impact availability of arginine [49,50].

Mometasone was investigated by Glassberg et al. based on an intuitive and transitive relationship that has been observed with pulmonary pathological-obstructive symptoms and their potential contribution to SCD VOCs [26]. Put simply, inflammatory markers, ventilation-perfusion mismatch, increased airway resistance, and hyper-reactivity have commonly been observed as characteristics of VOC episodes [51]. The logic then stands that by treating significant contributory symptoms of the VOC with known effective drug therapy, the episodes themselves can perhaps be reduced. This premise, however, is largely without precedent. In fact, in the Glassberg et al. paper reviewed here, the authors state that to their knowledge there have been no other papers published in the area of asthma medication used for treatment of SCD [26]. It is essentially a proof-of-concept study to set the stage for larger, more robust multicenter trials and perhaps even regulated indication for use. Experience has taught that the repurposing of drugs and identifying novel, expanded indications for existing drugs can lead to extraordinary clinical value. While the more popular examples include sildenafil, botox (onabotulinumtoxin A), and adalimumab, there have actually been many more. In fact, of the 197 drugs that became generic between July 1997 and May 2020, 32% of them had at least one new indication added [52]. The approach conceptualized by Glassberg et al. for inhaled steroids, therefore, has merit indeed.

Their mometasone study is unique in that not only did they report improved clinical outcomes, but they also measured biomarkers in order to support the mechanism [26]. A total of 35 non-asthmatic patients were randomized on a 2:1 basis resulting in enrollment of n=17 (placebo) versus n=35 (treatment). The soluble vascular cell adhesion molecule (sVCAM) marker on a percentage basis was observed as +8.82% for placebo versus -4.86% for mometasone. sVCAM-1 is known to be correlated with hypertension, vascular tissue inflammation, and endothelial dysfunction systemically [53]. Additionally, change in reticulocyte percentage was favorable for the mometasone arm.

In terms of limitations, the most important was that the trial size was small, although this design was intentional. It was also conducted at a single site. The authors reference the all-important Unverzagt publication [54], which demonstrates how trials conducted at a single site bias toward larger treatment effect estimates. Finally, for reasons that were not measured or further understood, the mometasone group demonstrated lower adherence to the treatment regimen. Obviously, this compliance bias would suggest an intention-to-treat (ITT) prejudice toward the null hypothesis. Ultimately, however, the objective was accomplished in favorably proving the steroid hypothesis to set the stage for larger, more robust trials.

Vitamin D represents an interesting therapeutic candidate. It is the only treatment reviewed that included both favorable [7] and unfavorable [8] outcomes. In a similar fashion to mometasone, the vitamin D hypothesis is built on an observation of SCD patient signs and symptomology, and not necessarily on a de novo mechanism of action. It has been established that a disproportionate percentage of SCD patients exhibit vitamin D deficiency, with some published estimates putting the prevalence as high as 96% [55]. As the logic goes, by treating such a profound sign, it may be possible to back into an effective curative mechanism. While the exact mechanism and etiology are acknowledged as unknown, it has been proposed that the nephropathy resulting from SCD leaves the kidneys unable to metabolize vitamin D [56].

Gregoire-Pelchat et al. measured both biomarkers and clinical outcomes with vitamin D [8]. Two regimens were deployed toward raising 25-hydroxyvitamin D [25(OH)D]. Patients ages five to 17 years were randomized to either a treatment regimen (n=18) receiving both a daily dosage of 1,000 IU (International Units) and a 300,000 IU bolus, or a placebo group (n=20) receiving only the 1,000 IU daily dose. The rise in 25(OH)D levels for the treatment group were impressive indeed: nearly a 10x increase, with statistical significance (20 ± 16 nmol/L treatment versus 2 ± 19 nmol/L placebo, p=0.003).

The metabolism of vitamin D is complex. There are three key steps. First is 25-hydroxylation, followed by 1α-hydroxylation, and, finally, 24-hydroxylation. All steps are catalyzed via cytochrome P450 mixed-function oxidases (CYPs). CYP2R1 is located in the endoplasmic reticulum (ER); CYP27A1, CYP27B1, and CYP24A1 are found in the mitochondria [57]. Efficacy associated with such a significant loading dose suggests that saturation of these metabolites can overcome the pathology.

Beyond the favorable increase in markers, however, Gregoire-Pelchat et al. were not able to demonstrate significant differences between treatment versus control for quality of life parameters, pain scores, or other clinical benefits including number of hospital days and number of treatment facility visits. Given that no clinical benefits were reported despite the fact that 25(OH)D levels crossed above 75 nmol/L in 83% of the treatment participants versus only 45% of the placebo participants calls into question how direct the linkage between these markers and clinical benefit may actually be.

To the contrary, Dougherty et al. were successful in putting together a regimen that showed superiority with vitamin D in type SS sickle cell disease (HbSS) patients for pain score reduction [7]. Of note is the absence of bolus dosing in their study design. They simply randomized HbSS and healthy patients into one of two regimens: 4,000 versus 7,000 IU vitamin D oral daily dosage. An exemplary outcome was that the baseline, six-week, and twelve-week pain scores for HbSS patients (PROMIS pain impact t-score) decreased from 54.4 to 52.7 to 48.4, respectively. From baseline until the last measurement, that represents a decrease of more than 10%. As one would expect, the trend for improved biomarker levels was also striking. For HbSS patients enrolled in the 4,000 IU arm, 25(OH)D increased from 18.0 to 38.1 over the course of 12 weeks, while the 7,000 IU arm experienced an impressive increase over the same time period from 20.8 to 53.1, or more than two times. These findings of Dougherty et al. are supported by other meta-analysis findings that demonstrate decreased pain scores and improved pain in chronic fibromyalgia pain patients [58].

The final treatment reviewed in this analysis that resulted in favorable outcomes was GLN. It is interesting that two of the five beneficial therapies are amino acids (arginine, glutamine). Niihara et al. published findings on glutamine from a phase 3 multicenter RCT [5]. The glutamine treatment group (n=152), when compared to the matched placebo group (n=78), showed fewer pain crises (3.0 vs. 4.0, p=0.005) as well as fewer hospitalizations (2.0 vs. 3.0, p=0.005) over the course of 48 weeks. It should be noted that two-thirds of the patients in each group received concomitant hydroxyurea. The RCT reviewed here was funded and executed on the heels of a successful phase 2 clinical trial in which glutamine was shown to be superior to placebo over a six-month duration for reducing VOCs and hospitalizations [59].

Glutamine is required to synthesize nicotinamide adenine dinucleotide (NAD, or NADH in its reduced form), which acts as a ubiquitous oxidation-reduction (redox) cofactor in RBCs. Uptake of glutamine in sickle cells has been reported as several times greater than in normal RBCs [60]. The result of glutamine administration is improved maintenance of the [NADH]:[NAD+ + NADH] redox balance and therefore decreased oxidative stress. Finally, sickled RBCs in patients taking glutamine have demonstrated significantly less adherence to umbilical vein endothelial cells [61].

Endari (glutamine) approval was granted in July 2017 to Emmaus Medical, the company listed in the disclosures of the Niihara et al. as supporting the study [62]. It is currently available in powder form at a recommended dose of 10-30 g daily based on body weight. Each dose is mixed with 8 oz. or 240 mL of cold or room temperature beverage [63]. Common side effects listed on the label for use include constipation, nausea, headache, cough, abdominal pain, and pain in the extremities, back, and chest [64].

In terms of analyzing the research literature by comparing the five proven effective treatments (crizanlizumab, mometasone, arginine, vitamin D, glutamine) against one another, it is a difficult endeavor for several reasons. First, there were multiple endpoints used to define effective versus no difference compared to control. Second, even within the same endpoint, there were differences. For example, multiple indices were used to measure pain scores. Additionally, even though all trials included were RCTs, there were wide differences from publication to publication in terms of design, ranging from simple n=44 patient pilot studies [7] to robust n=230 phase 3 clinical trials for FDA clearance [5], and to others featuring 66 locations, 12 countries, and 60 cities [26]. Finally, not all of the trials were controlled in the same fashion. Dougherty et al. structured the treatment and control arms as comparative therapeutic doses of vitamin D as opposed to using a placebo [7], and Niihara et al. utilized concomitant hydroxyurea to both the treatment and control arms in their investigation of glutamine [5].

It will be of great utility to have investigations that combine different therapies with different mechanisms of action into a single treatment plan. This practice of drug "cocktails" is commonplace in the treatment of cancer. Gilad et al. concluded that the concept of combinational chemotherapy drug treatment, which was initially begun as a mechanism for overcoming drug resistance, is now a standard practice [65].

Limitations

The decision was made to exclude any form of publication outside of RCTs. Other publication forms such as case reports, case series, retrospective studies, and observational studies were not included and may have been valuable.

SCD VOC is a complicated clinical presentation. While the aim was to focus on pain management publications (reflected in the keyword strategy), it is possible that research literature exists that investigates this patient profile but without specifically referencing it as such. Put in analogous diagnostic lingo, this review features high specificity but perhaps low sensitivity.

And, finally, the most significant limitation is the lack of consistency of endpoints from one trial to the next. This lack of uniformity not only makes meta-analysis of data impossible but also prohibits the difference of means and standard deviation analysis.

## Conclusions

The evidence evaluated in this review supports a number of emerging therapies as having the potential to reduce the intensity and duration of VOCs in SCD patients. A total of 17 discrete options/candidates for treatment were identified. Our methodology only looked back five years to 2017, and therefore did not include traditional mainstay therapies such as hydroxyurea, for which no new RCTs have been published in that time.

It will be of great value moving forward to have research that combines different therapies with different mechanisms of action into a single treatment approach. The question becomes, for example, would a patient treated with both crizanlizumab and glutamine therapy together benefit versus using just one drug or the other? Furthermore, it is striking that given such an extensive list of candidates, only a small number were proven to be beneficial, with an even smaller number still demonstrating evidence convincing enough to justify clearance for use by regulating authorities.

Finally, it is less than encouraging that the correlation between biomarkers and clinical outcomes is not more direct and positive. This review analyzed several studies in which there were beneficial outcomes observed for increasing target biomarkers, yet in these same investigations, such cellular and molecular mechanisms of action did not translate into statistically significant clinical endpoints with practical importance.

SCD is a terrible disease and VOCs are its most dreaded complication. While the list of proven efficacious therapies is currently short, there is a preponderance of encouraging research that should lead to the development of superior solutions with time and understanding.
